# Coronin7 regulates WASP and SCAR through CRIB mediated interaction with Rac proteins

**DOI:** 10.1038/srep14437

**Published:** 2015-09-28

**Authors:** Karthic Swaminathan, Maria Stumpf, Rolf Müller, Anna-Carolin Horn, Julia Schmidbauer, Ludwig Eichinger, Annette Müller-Taubenberger, Jan Faix, Angelika A. Noegel

**Affiliations:** 1 Center for Biochemistry, Medical Faculty, Center for Molecular Medicine Cologne (CMMC) and Cologne Excellence Cluster on Cellular Stress Responses in Aging-Associated Diseases (CECAD), University of Cologne, 50931 Köln, Germany; 2 Institute of Anatomy and Cell Biology, Ludwig-Maximilians-University, 80336 München; 3 Department of Biophysical Chemistry, Medizinische Hochschule Hannover, 30623 Hannover, Germany

## Abstract

Coronin7 (CRN7) stabilizes F-actin and is a regulator of processes associated with the actin cytoskeleton. Its loss leads to defects in phagocytosis, motility and development. It harbors a CRIB (Cdc42- and Rac-interactive binding) domain in each of its WD repeat domains which bind to Rac GTPases preferably in their GDP-loaded forms. Expression of wild type CRN7 in CRN7 deficient cells rescued these defects, whereas proteins with mutations in the CRIB motifs which were associated with altered Rac binding were effective to varying degrees. The presence of one functional CRIB was sufficient to reestablish phagocytosis, cell motility and development. Furthermore, by molecular modeling and mutational analysis we identified the contact regions between CRN7 and the GTPases. We also identified WASP, SCAR and PAKa as downstream effectors in phagocytosis, development and cell surface adhesion, respectively, since ectopic expression rescued these functions.

Coronins are a large family of evolutionary conserved proteins. They consist of a WD (Tryptophane- Aspartate)-repeat domain containing seven repeats that form a seven-bladed β-propeller structurally resembling the Gβ subunit of the heterotrimeric G-proteins. This region is followed by a unique region and a C-terminal coiled coil region which mediates oligomerization. Coronins play roles in actin cytoskeleton-associated processes, in signal transduction, in endosomal trafficking, survival of pathogenic bacteria in macrophages and homeostatic T cell signalling^1^,^2^,^3^. In addition to coronins with one propeller, there exist coronins with two WD-repeat containing regions, the coronin7 proteins, and coronins which are composed of an N-terminal WD-repeat followed by PH domains fused to villin^4^. *Dictyostelium discoideum* harbors a conventional coronin, the product of the corA gene, a coronin7 (CRN7) encoded by corB and villidin with PH and gelsolin/villin domains^4^,^5^. Mutant analysis revealed roles for the coronin proteins in phagocytosis, chemotactic motility and development^6^,^7^.

Little is known how coronins are controlled. A recent publication put coronin downstream of the receptor(s) activated by secreted factors and upstream of the activation of the cAMP relay but how coronin activation was achieved was not addressed^8^. We studied a Cdc42/Rac interactive binding (CRIB) motif in coronin and its interaction with small GTPases of the Rac family. The coronin CRIB was originally identified in *Xenopus* coronin. Its biological significance was revealed in expression studies where active Rac caused relocalization of coexpressed coronin to the cell periphery^9^. The authors of this study also showed that the CRIB related motif could interact with and recruit Rac. Subsequently they were identified in all mammalian coronins and in the *Dictyostelium* coronins. A minimal CRIB motif encompasses ~15 amino acids and is divided into two halves which are separated by a region of variable length. In a core of eight amino acids (ISXPXXXXFXHXXHVG) one to two substitutions are tolerated. In the coronins it is located at the surface of the β-propeller between blades 2 and 3.

Rac proteins are important regulators of the actin cytoskeleton and their activation leads to the assembly of contractile actin myosin filaments and formation of actin rich protrusions^10^. We found that coronin through its CRIB domain bound Rac proteins preferentially in their GDP-loaded form. Upon loss of coronin the levels of activated Rac increased in cells and led to PAKa activation. Activated PAKa phosphorylates myosin heavy chain kinases thereby inactivating them. They can no longer phosphorylate myosin II which would lead to myosin filament disassembly. This mechanism explains the observed myosin II overassembly in the *corA*^−^ strain^11^.

Here we analyze the Rac binding activity of coronin7. In *D. discoideum* coronin7 is associated with the actin cytoskeleton. It binds directly to F-actin and stabilizes the filaments preventing their disassembly. Mutants lacking the protein exhibit enhanced phagocytosis of yeast particles, defective cell substratum adhesion, motility and enhanced development letting us conclude that coronin7 has an inhibitory impact on these processes^7^,^12^. This inhibitory action could be achieved through the direct F-actin interaction and/or through controlling F-actin dynamics by affecting cytoskeletal regulators like Rac proteins and their effectors.

## Results

### The CRIB motifs of coronin7

**Figure 1:**
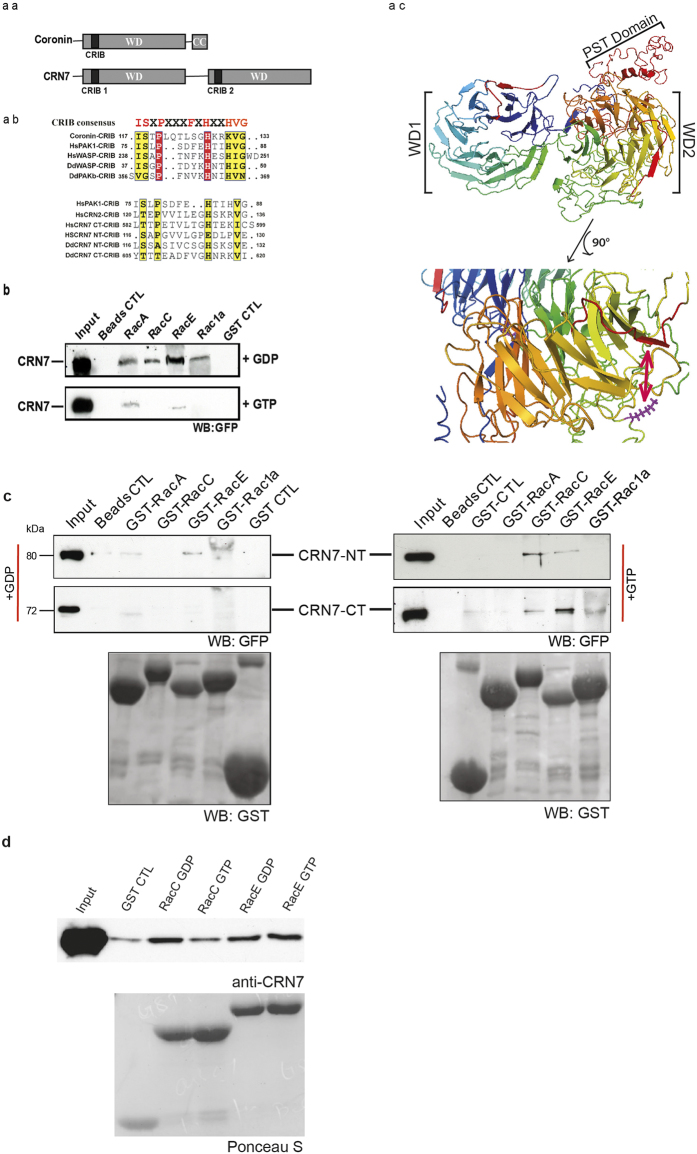
The CRIB domains of CRN7 and Rac GTPase interactions of CRN7. (aa) Domain structure of coronin and CRN7. (ab) Sequence alignment of the CRIB domain in coronin and CRN7 and selected proteins. The consensus sequence is shown above the alignment. The position within the proteins is indicated. (ac) Model of CRN7. The 3D structure was predicted as described^11^. The CRIB domains are in shown red. A magnification of the region containing the CRIB domain and the putative F-actin binding site in WD2 is shown below. The conserved K residue is indicated in a stick model in magenta. (**b**) CRN7 interacts primarily with GDP-bound Rac. GST and GST-Rac proteins loaded with GDP or GTPγS were bound to Glutathione Sepharose beads and incubated with lysates of *corB*^−^ cells expressing GFP-CRN7 WT. The bound proteins were analyzed by western blotting. Probing was with GFP specific mAb K3-184-2. (**c**) The N- and the C-terminal half of CRN7 interact with Rac GTPases. The precipitated proteins were detected with mAb K3-184-2, the GST-fusions with polyclonal GST-specific antibodies. GST control (GST CTL) and beads control (Beads CTL) are shown. (**d**) CRN7 interacts directly with Rac proteins. Bacterially expressed GST-CRN7-NT encompassing the PST-domain was cleaved from the GST part and used in pull down assays with GST-Rac GTPases. The precipitated proteins were analyzed in western blots. CRN7-NT was detected with mAb K67-31-5.

Coronin7 (CRN7, DDB0232260^13^) harbors a CRIB motif in each of its beta propellers (Fig. 1aa–c). They are located at positions 116 to 131 (NT-CRIB) and 605 to 619 (CT-CRIB) between blades 2 and 3 of each β propeller (arrow in Fig. 1ac). A comparison with CRIB domains from other proteins shows that they are only moderately conserved (Fig. 1ab). The highlighted residues in the consensus CRIB domain ISXPXXXXFXHXXHVG are those that interact with the GTPases^14^. Of these only four positions are conserved in the CRIB domains of CRN7 (Fig. 1ab). Structural analysis of CRIB domains in complex with GTPases, however, revealed that there can be some variation. Furthermore, adjacent sequences also contribute to the binding, and less well conserved CRIB domains lead to successful GTPase interactions.

*D. discoideum* harbors several GTPases of the Rac family^15^. We expressed several of them as GST-fusion proteins, preloaded them with GDP or GTPγS and used them to pull down GFP-tagged CRN7 from cell lysates and probed the presence of GFP-CRN7 with GFP specific mAb K3-184-2. All of the tested Rac GTPases (A, C, E, 1a and 1b) interacted with GFP-CRN7. It was however the GDP loaded form of the proteins which preferentially precipitated GFP-CRN7 (Fig. 1b and data not shown). GFP-CRN7 did not bind to GST coated beads or beads only revealing the specificity of the binding (Fig. 1b,c). In order to study the binding of Rac to the individual WD repeat domains of CRN7, we expressed the two domains of CRN7 separately as GFP fusions (GFP-CRN7-NT and GFP-CRN7-CT^12^) and found that in general both domains preferred the GDP loaded form of the Rac GTPases. An exception was seen for the GFP-CRN7-CT RacE interaction where GFP-CRN7-CT showed a preference for GTP loaded RacE (Fig. 1c). In order to probe whether the binding is direct we used bacterially produced CRN7-NT-PST which was cleaved from the GST-tag and carried out pulldown expriments with GST-Rac proteins. GDP- and GTPγS-loaded RacC and RacE precipitated the recombinant polypeptide which was detected with CRN7 specific mAb K67-31-5 (Fig. 1d).

Since CRN7 binds primarily to Rac GTPases in their GDP-bound form it might influence the levels of active Rac proteins and a loss of CRN7 could lead to enhanced levels of GTP-loaded Racs. When we tested this in the CRN7 deficient strain (*corB*^−^) by performing pull-down assays with the GST-tagged p21 binding domain of PAK (GST-PBD) from rat which interacts with activated Racs in cell lysates we found however no significant alteration in the levels of activated Rac in AX2 and *corB*^−^ (data not shown). These results contrast with the findings in the *corA*^−^ mutant where we observed increased levels of activated Rac. This could be due to the fact that coronin is a rather abundant protein^16^. In fact, when we compared transcript levels for coronin and CRN7 in mRNA from growth phase cells by qRT-PCR we found threefold higher levels for coronin. Furthermore, a direct comparison of coronin and CRN7 showed that coronin binding to Rac proteins is more efficient (Supplementary Figure S1).

### Impact of mutations in the CRIB motifs on Rac binding

**Figure 2:**
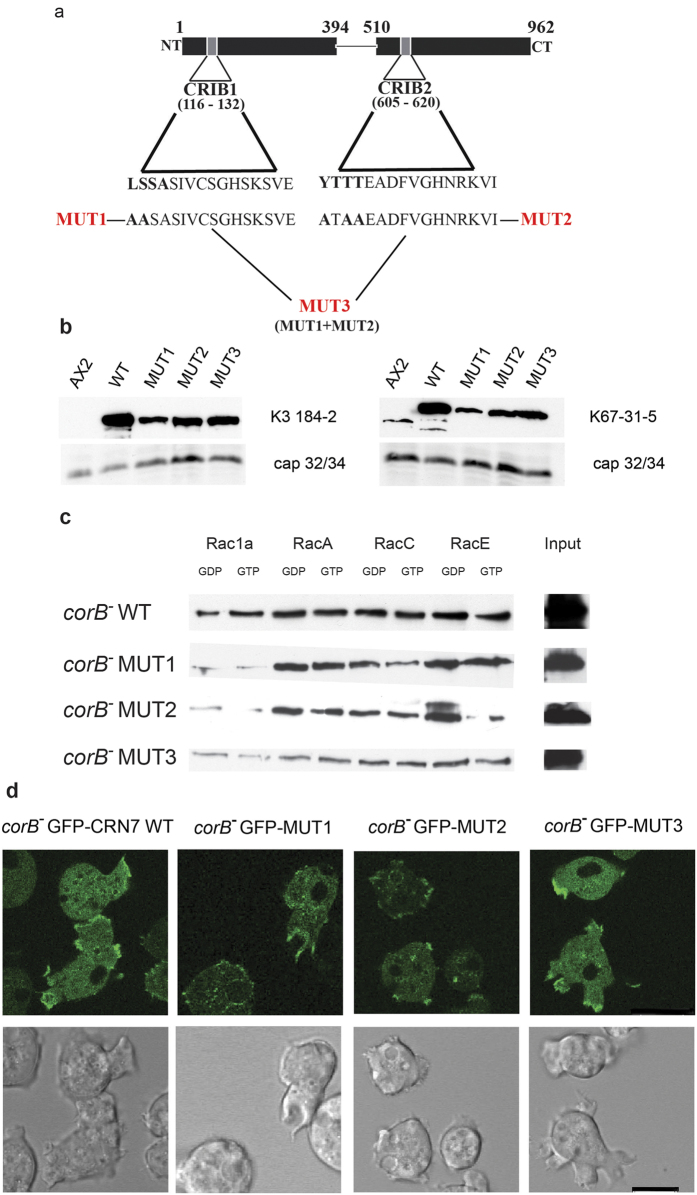
Mutations of the CRN7 CRIB domains affect Rac GTPase binding. (**a**) Generation of the CRN7 CRIB mutants. The proteins were expressed as GFP fusions in *corB*^−^. (**b**) Expression levels of the mutant protein. Cell homogenates from equal numbers of cells were analyzed by western blotting. Probing was with mAb K67-31-5 which detects CRN7 and mAb K3-184-2 recognizing the GFP-tagged proteins. For loading control cap34 was detected by mAb 135-409. (**c**) Interaction of CRN7 WT and CRIB mutant proteins with Rac GTPases loaded with GDP and GTPγS and bound to Glutathione-Sepharose beads. The bound proteins were immunoblotted and probed with mAb K3-184-2. I, input, aliquot of the cell lysate used in the assay. (**d**) Live cell analysis of GFP-CRN7 WT and GFP-CRN7 CRIB mutants. Representative images together with the corresponding differential interference contrast images are shown. Scale bar, 10 μm.

The CRIB motifs CRIB1 in the N-terminal β-propeller and CRIB2 in the C-terminal β-propeller are thought to be surface accessible based on their location between blades 2 and 3 of the respective β-propeller domains (Fig. 1ac). To assess the significance of the individual CRIB motifs we generated point mutations and changed the N-terminal conserved residues of CRIB1 from LSSA to AASA and of CRIB2 from YTTT to ATAA by site directed mutagenesis in a plasmid encoding GFP-CRN7 WT thereby generating GFP-MUT1 and GFP-MUT2, respectively. In GFP-MUT3 both CRIB domains were mutated (Fig. 2a). Expression was under the control of the constitutively active actin15 promoter^12^. The plasmids were transformed into *corB*^−^ cells for analysis of the Rac binding activities of the proteins and of the significance of the CRIB motifs for the function of the protein. The levels of expression differed for the mutant proteins. CRN7 WT was strongly overexpressed, MUT1, 2 and 3 levels were also higher as compared to the one of the endogenous protein in AX2 (Fig. 2b).

In the Rac binding assays we used GST-tagged Rac1a, Rac1b, RacA, RacC and RacE preloaded with GDP or GTPγS for pull down experiments and probed the precipitates with GFP specific antibodies for the presence of CRN7 WT and mutant proteins. Whereas the wild-type protein had shown a stronger interaction with the GDP-bound Rac proteins, the mutant proteins behaved differently and had lost their preference in several cases. In our pulldown assays, differences were observed in the binding affinities to various *Dictyostelium* Rac family members. In general, WT and CRIB mutant proteins bound stronger to the GDP-loaded forms of the Rac sub-family members RacA, RacC and RacE compared to the Rac1 family members Rac1a and Rac1b (Fig. 2c; Supplementary Figure S2a). However, we noted that all the mutant proteins lost their preference for the GDP-loaded form of specific Rac family member proteins. MUT1 still preferred the GDP versions of Rac1b and RacC, but has lost preference in case of Rac1a, RacA and RacE and interacted equally well or slightly better with their GTPγS-loaded forms (Supplementary Figure S2b and data not shown). MUT2 preferred GDP-loaded Rac1a and RacE, for Rac1b, RacA and RacC we noted no preference for the GDP-loaded forms. MUT3 preferred GTPγS loaded Rac1b and RacC and showed no preference in case of Rac1a, RacA and RacE. Also, binding was reduced (Fig. 2c; Supplementary Figure S2b). We conclude from this data that the CRIB motifs (CRIB1 and CRIB2) of CRN7 show general binding ability for all Rac family members in the GDP-bound form. Taken together, mutation in the CRIBs affects the preference to only a sub-set of Rac family members. In case of MUT3 it also resulted in reduced overall binding ability (Fig. 2c and data not shown). In control experiments none of the CRN7 proteins showed binding to GST or beads only (data not shown).

We also wondered whether the CRIB mutations alter the F-actin binding ability of the proteins. An F-actin binding site has been identified near the N-terminus of Coronin 1A and 1B. An essential arginine (R31) is located between blades 7 and 1 and is conserved in *Dictyostelium* coronin and CRN7 where a lysine replaces the arginine^17^. The locations of the lysines are indicated in Fig. 1ac (in magenta). The CRIB domains are further away between blades 2 and 3 of the β-propeller and an influence of the mutations on F-actin binding appears not very likely. Moreover, when we tested whether the mutant proteins retain their ability to associate with the actin cytoskeleton we found all proteins in the Triton-insoluble fraction as reported for CRN7 previously^12^ (Supplementary Figure S3). Also, when we analyzed the distribution of the mutant proteins in living cells we found that the wild type and CRIB mutant GFP fusion proteins behaved nearly identical and were present throughout the cytosol and redistributed in a reversible manner to the plasma membrane where they accumulated at newly forming extensions like pseudopods and macropinosomes and on intracellular large vesicular structures (Fig. 2d).

### RacC mutational studies reveal the contact region with CRN7

**Figure 3:**
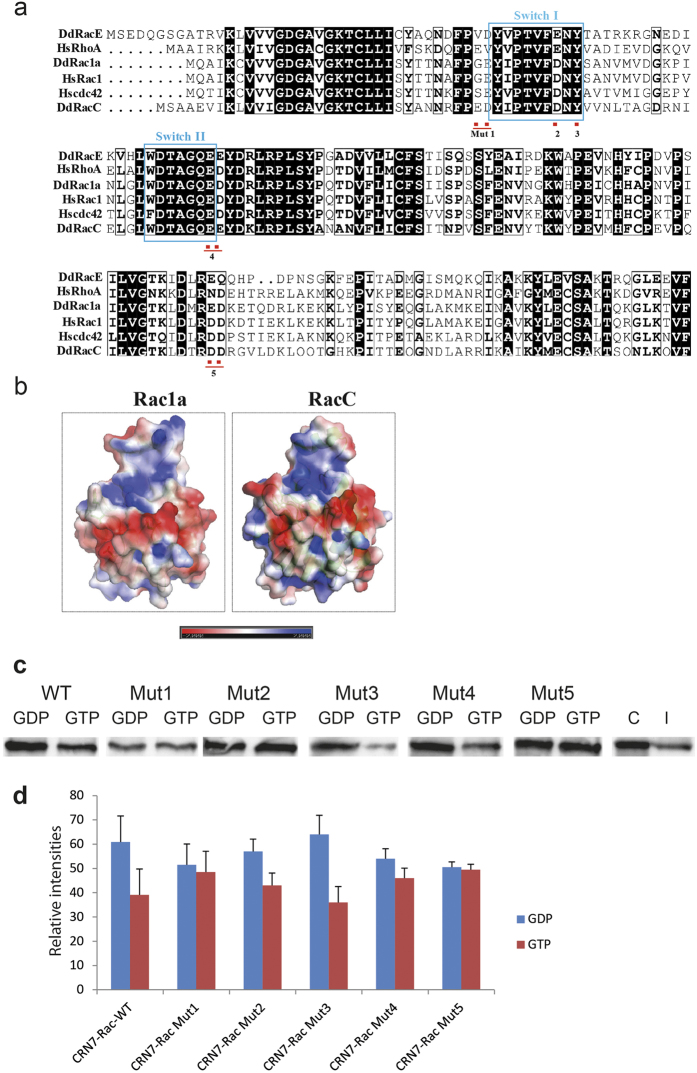
Mutational analysis of RacC. (**a**) Alignment of various Rac proteins. The position of the mutations generated is indicated as well as of the Switch I and II regions. (**b**) Surface potential of *D. discoideum* Rac1a and RacC. The three dimensional structure of Rac1a and RacC is modeled using SWISSMODEL and the surface potential calculated using APBS plug-in in Pymol. Red, negative, blue, positive charge. (**c**) Interactions of RacC Mut1-5 with GFP-CRN7 WT. The RacC mutant proteins were expressed as GST fusions, bound to Glutathione-Sepharose beads, loaded with GDP or GTPγS and used to pull down GFP-CRN7 WT from cell lysates. C, represents the protein amounts in 2 × 10^5^ cells; I, aliquot of the cell lysate used in the assay. The western blots were probed with mAb K3-184-2. (**d**) Binding affinities of different Rac mutants to GFP-CRN7. Bar chart shows the binding of RacC wild type and mutant proteins loaded with GDP or GTPγS to GFP-CRN7. Between three and five experiments were carried out. Relative intensities are shown as arbitrary units ± SD. The differences for wild type RacC (WT), Mut2 and Mut3 were statistically significant (P < 0.02, 0.0015 and 0.01, respectively).

Next, we set out to map the binding interface of CRN7 on Rac GTPases. In our binding assays, we have found that CRN7 showed varying binding affinities to different Rac members, higher for RacE and less pronounced for Rac1a (Fig. 1b). In order to understand how CRN7 recognizes different Rac family members, we first aligned the protein sequences of *D. discoideum* Rac members with human Rac, Rho, and Cdc42 and looked into the differences in the primary sequences. All of the *D. discoideum* Rac members showed conserved Switch I and Switch II regions comparable to mammalian GTPases (Fig. 3a). However, there are subtle variations observed in the amino acid composition of regions around the Switch I and Switch II. For instance, all Rac members, both mammalian and *D. discoideum*, showed a conserved aspartic acid at position 38 but it was mutated to glutamic acid only in RacE and human RhoA. We next asked if this difference reciprocates in the three dimensional structure of the proteins by modeling the structure of *D. discoideum* RacC and Rac1a using mammalian Rac1 as a template. We calculated the surface potential of these proteins using Pymol APBS plug-in. Comparison of RacC and Rac1a structures showed a major difference in the surface properties of these proteins which they present into the outside environment (Fig. 3b). In order to determine if these differences govern the binding affinities of Rac to CRN7, we created two sets of mutations in the RacC protein. The first set of charge reversal mutations targeted the surface residues around the Switch regions that showed differences among the Rac family members (Fig. 3a). These mutations included charge reversals (Mut1, ED to KK, and Mut4, EE to KK), introduction of a hydrophobic residue instead of a charged one (Mut2, D to A), and polar residues instead of charged residues (Mut5, DD to QQ). In addition, previous work has shown that all of the interacting partners of the Rho GTPases including those with a CRIB domain bind to a common set of conserved amino acids that are clustered on the surface of the Switch regions^18^. This includes a conserved Tyrosine at postion 40 which is recognized by all CRIB family member proteins. We have replaced this Tyrosine with a charged residue in our Mutant 3. In short, Mut1, 2 and 3 are located next to or in Switch 1. In Mut3 an essential Tyrosine in Switch 1 is targeted, Mut4 is located at the end of Switch 2 and the residues changed in Mut5 precede the α3b-helix towards the C-terminus (Fig. 3a). Switch 1 and Switch 2 are those regions in the Rac GTPases which change their conformation upon GDP or GTP binding.

The RacC mutants were expressed as GST fusion proteins in *E. coli*, purified and used to pull down GFP-CRN7 WT from AX2 cells. All mutant proteins interacted with GFP-CRN7 WT and precipitated the protein in their GDP and the GTPγS loaded form which suggests that the mutations do not disrupt binding of CRN7. Both forms of Mut1 were nearly equally efficient in the pull down experiment. Mut2 and Mut3 were more efficient in their GDP-loaded form and behaved like wild type RacC, Mut4 was slightly more efficient in its GDP-loaded form, and both forms of Mut5 precipitated CRN7 equally well (Fig. 3c,d). Furthermore, the interaction between GFP-CRN7 WT and Mut1 appeared to be weaker. We conclude from our results that contacts with CRN7 are made in regions of Switch1 and also outside of the Switch regions (Mut5) in RacC (see Discussion).

### Impact of CRIB mutations in CRN7 proteins on cellular properties

In our previous studies we observed in our analysis of CRN7 deficient cells that the protein had an impact on the dynamics of the actin cytoskeleton, affected phagocytosis, motility and development, and we had concluded that CRN7 acted as an inhibitor. Here we studied which mutant protein can rescue these defects in *corB*^−^ cells. This should indicate which one of the functions depends on the interaction of CRN7 with Rac proteins and is linked to a particular Rac binding site. We also included cell substrate adhesion assays since we found this property significantly altered in the mutant.

**Figure 4:**
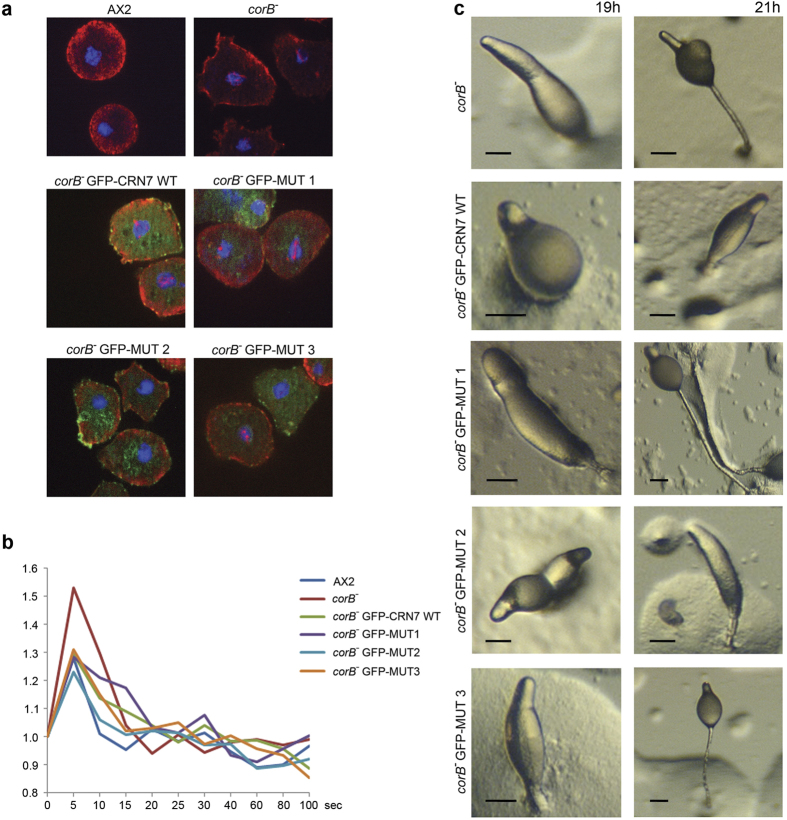
Expression of GFP-CRN7 WT and mutant proteins rescues defects in *corB*^−^. (**a**) Analysis of DMSO induced actin rod formation in the nuclei of *corB*^−^ and *corB*^−^ expressing WT and mutant CRN7 as indicated. Cells were stained for actin with mAb act1 and DAPI to visualize the nucleus. Cells expressing comparable amounts of GFP-tagged CRN7 proteins were chosen for the analysis. (**b**) cAMP induced F-actin accumulation. Cells were starved for six hours and then stimulated with a single pulse of cAMP. Triton insoluble cytoskeletons were prepared and the F-actin content determined using TRITC phalloidin. The data shown represent the mean values of between four to eight experiments. Error bars were omitted for clarity. (**c**) Development of *corB*^−^ and *corB*^−^ expressing GFP-CRN7 WT and mutant proteins. Cells were starved on phosphate agar plates (5 × 10^7^ cells per 10 cm dish). Pictures from the 19 and 21 h time point are shown. Representative developmental structures are shown. Scale bar, 50 μm.

To study the dynamics of the actin cytoskeleton we followed the response to DMSO treatment. DMSO leads to a reversible disassembly of the actin cytoskeleton and the formation of actin rods in the nucleus. In *corB*^−^ cells the cortical actin disappeared faster and nuclear actin rods had formed earlier than in AX2^7^. Expression of GFP-CRN7 WT and MUT1 enhanced the sensitivity towards DMSO and the cells harbored more nuclear actin rods than the *corB*^−^ cells at the 10 minutes time point (45% *corB*^−^ cells with rods, 52% of GFP-CRN7 WT and 54% of MUT1 cells with rods). MUT3 resembled AX2 cells (25% for MUT3 and 30% for AX2), and in MUT2 actin rod formation was less pronounced (13% of cells with actin rods at the 10 minutes time point). (Fig. 4a, Table 1).

F-actin changes also occur in response to a hormone stimulus. Upon application of cAMP a characteristic pattern of actin polymerization and depolymerization occurs with an initial increase of F-actin at the 5 sec time point followed by depolymerization and a longer lasting phase of polymerization. In the *corB*^−^ strain the initial F-actin peak was consistently higher than in AX2^7^. Expression of GFP-CRN7 WT and all MUT proteins led to a similar reduction in the height of the first peak and reestablished wild type levels. This data suggests that CRN7 mediated regulation of actin polymerization in response to a stimulus is independent of its Rac binding ability (Fig. 4b).

The presence of CRN7 in a cell has an inhibitory impact on phagocytosis since *corB*^−^ cells are much more active in uptake of yeast particles^7^. Expression of GFP-CRN7 WT reduced the uptake activity to wild type levels as did MUT1 and MUT2 whereas MUT3 expressing cells behaved like the *corB*^−^ mutant (Table 1). In the microscopic analysis we tried to evaluate only those cells which expressed similar amounts of the proteins to exclude possible effects of overexpression although previous experiments had shown that overexpression of GFP-CRN7 WT does not have an impact on phagocytosis in wild type AX2 cells^12^. It appears that for phagocytosis a single intact CRIB domain ensures the functioning of the protein in phagocytosis.

Motility could be a further aspect in which CRN7 is involved. In our previous analysis of chemotactic motility we had observed a slight but not significant reduction in the *corB*^−^ strain^7^. When we analysed random motility of vegetative cells, *corB*^−^ cells showed a slightly reduced speed (2.87 ± 1.28 μm/min) as compared to AX2 (4.46 ± 2.52 μm/min) although the difference, again, was not significant. Expression of GFP-CRN7 WT, MUT1 and MUT2 improved motility towards wild type, whereas MUT3 did not (Table 2). As seen for phagocytosis a single intact CRIB ensures cell motility (Supplementary Table 1).

Adhesion normally depends on cell surface proteins and downstream molecules such as proteins of the Sec7 family of ArfGEFs or paxillin which link the cell surface protein to the cytoskeleton^19^. Upon analysis of the cell substratum adhesion we found that *corB*^−^ cells adhered significantly stronger to a plastic surface than AX2. Expression of GFP-CRN7 WT did not affect the adhesion behavior of *corB*^−^ whereas MUT1 and MUT2 showed some improvement and were less adhesive, although the difference was not significant. The absence of a rescue by GFP-CRN7 WT could be the result of the overexpression (Table 1).

The *corB*^−^ strain has an accelerated development from aggregation onward and development is completed with the formation of fruiting bodies approximately four hours earlier than in AX2^7^. Developmental timing of MUT1 and MUT3 expressing strains was similar to *corB*^−^ whereas development of GFP-CRN7 WT and MUT2 was slower and resembled the behavior of AX2. For all strains development ended with the formation of normal looking fruiting bodies (Fig. 4c).

The data presented above can most likely be associated with the CRIB domains and their impact on GTPase binding and less so with the direct F-actin interaction of CRN7 since we did not observe an altered cytoskeletal association (Supplementary Figure S3).

### Downstream effectors

#### CRN7 regulates phagocytosis through WASP inhibition

There exist several effectors that connect activation of Rho-GTPases with cytoskeletal targets. Prominent examples are WASP (Wiskott-Aldrich syndrome protein), SCAR/WAVE, p21 activated kinase (PAK), formins and IQGAP. The most striking phenotype of CRN7 mutant cells is the increased phagocytic uptake^7^. Therefore we focused first on Rac effectors that play a key role in phagocytosis. WASP, the founding member of the WASP family proteins, has been implicated primarily in endocytotic processes both in *D. discoideum* and mammalian cells^20^. It functions by activating Arp2/3 complex mediated actin polymerization. In *D. discoideum* low levels of WASP resulted in reduced F-actin levels affecting chemotactic motility and development^21^,^22^.

**Figure 5:**
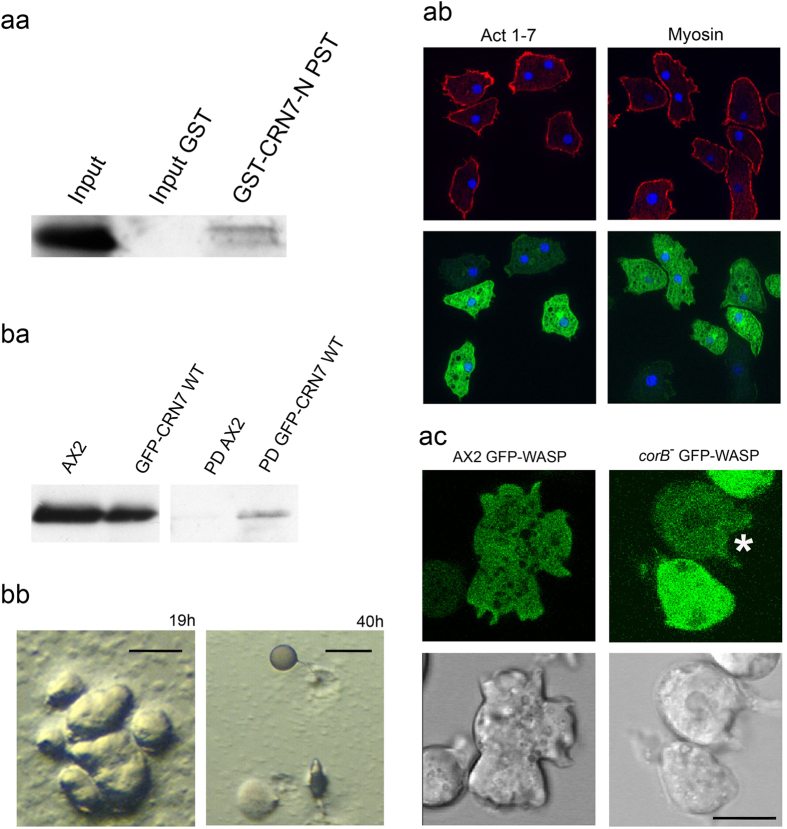
Downstream effectors of CRN7. (**a**) WASP as **a**n effector of CRN7. (aa) Interaction of WASP and CRN7 using GST fusions of CRN7. GFP-WASP was expressed in *corB*^−^ cells, GST-CRN7-N PST was used to pull down GFP-WASP (70 kDa). For control GST coated beads were used (Input GST). Detection was with mAb K3-184-2. (ab) Localization of GFP-WASP in *corB*^−^ cells. Cells were fixed with methanol and stained for actin (mAb act1) and myosin (mAb 56-396-5). (ac) Live cell analysis of GFP-WASP in AX2 and *corB*^−^. Representative images together with the corresponding differential interference contrast images are shown. Star points to GFP-WASP accumulation at a forming macropinoytic cup. Scale bar, 10 μm. (**b**) SCAR as effector. (ba) Interaction of SCAR and CRN7. GFP-CRN7 was precipitated using GFP-trap beads (PD), Scar (66 kDa) was detected with polyclonal antibodies. For control AX2 lysates were incubated with GFP-trap beads and the pull down probed for Scar (PD AX2). (bb) Development of *corB*^−^ expressing GFP-SCAR. Development was on phosphate agar plates. Images shown were taken after 19 and 40 hours. Scale bar, 250 μm.

Our attempts to show a direct interaction of CRN7 and WASP in GFP-WASP expressing AX2 cells were not successful although in pull-down assays using different GST-tagged CRN7 domains GFP-WASP was precipitated by GST-CRN7-N PST and to a lesser degree by the PST-domain (Fig. 5aa and data not shown). The GST control did not reveal non-specific binding of GFP-WASP.

Next we asked if WASP acts downstream of CRN7 by overexpressing GFP-WASP in *corB*^−^ cells and analyzing the effects of overexpression on F-actin levels, phagocytosis, cell substratum adhesion and motility. WASP has been found at the anterior leading edge and the uropod in chemotaxing cells where it is required for localized F-actin assembly^22^. In *corB*^−^ cells GFP-WASP was observed in the cytosol and at the cell cortex in fixed cells (Fig. 5ab). In living cells we observed that it was enriched in areas of the cell where protrusions were formed showing a similar behavior as GFP-CRN7 (Fig. 5ac).

F-actin levels and cell substratum adhesion were not significantly altered in the *corB*^−^ strain upon ectopic expression of GFP-WASP, whereas analysis of phagocytosis revealed a significant reduction and a complete return to wild-type levels suggesting that WASP acts downstream of CRN7 to regulate phagocytosis (Table 3; Supplementary Table 2). Cell motility did not differ significantly from the one of *corB*^−^ (Table 2) and development was as in the *corB*^−^ strain (data not shown). Altogether the data suggest that WASP is in a pathway located downstream of a CRN7-Rac complex that has an impact on phagocytosis.

### SCAR is a regulator of development and PAKa regulates adhesion

Next, we focused on SCAR, a known positive regulator of actin polymerization. In *D. discoideum* absence of SCAR resulted in increased speed of development reminiscent of that of *corB*^−^ cells. In addition SCAR^−^ strains displayed a mulitple-tip phenotype. Further, the cells had a smaller size than SCAR^+^ cells and the F-actin content was reduced^23^,^24^.

To assess if CRN7 interacts with SCAR, we performed co-immunoprecipitation assays using cells expressing GFP-CRN7 in which we found that SCAR could be co-immunoprecipiated with GFP-CRN7 using GFP-trap beads although the amounts in the precipitate were very low. Using GFP-trap beads with AX2 lysates did not bring down SCAR indicating that the binding was specific (Fig. 5ba).

Ectopically expressed GFP-SCAR did not show a particular enrichment in *corB*^−^ cells in live cell studies and was diffusely distributed in the cytosol (data not shown). F-actin levels were similar to those of *corB*^−^, yeast uptake was not altered nor was the cell substrate adhesion significantly affected (Table 3). It also did not have an impact on cell size. By contrast, GFP-WASP expressing cells showed a different size distribution and the majority of the cells had a diameter >12.1 μm. For *corB*^−^ and *corB*^−^ GFP-SCAR the majority had a diameter below 12.1 μm. (Table 3).

Both *corB*^−^ and SCAR^−^ cells showed a similar accelerated developmental phenotype. We first asked if there is a difference in the SCAR protein levels in vegetative *corB*^−^ cells compared to WT cells. We consistently observed mildly reduced SCAR protein levels in *corB*^−^ cells (AX2, 1.51 ± 0.75; *corB*^−^, 1.05 ± 0.43; relative amounts, six experiments). We hypothesized that the reduced SCAR levels observed in *corB*^−^ cells may contribute to the accelerated developmental phenotype observed in *corB*^−^ cells. To compensate the loss in SCAR protein levels, we overexpressed GFP-SCAR in *corB*^−^ cells and studied its effect on development. In the *corB*^−^ GFP-SCAR strain development was strongly delayed and tight aggregates were present at 19 hours when *corB*^−^ already had formed fruiting bodies. Culminants and fruiting bodies had formed only after 40 hours of development. The fruiting bodies had short stalks and big spore heads. Furthermore, during aggregation large aggregates were formed with multiple tips which gave rise to individual fingers and slugs (Fig. 5bb). The data suggest that SCAR is involved in the regulation of the developmental functions of coronin7.

To identify a player in adhesion we turned to PAK (P21 activated kinases). PAKs are further effectors of Rho GTPases carrying a CRIB domain. They are Ser/Thr protein kinases with the catalytic domain placed at the C-terminus and regulate actin dynamics and signal cascades involved in transcription, apoptosis and cell cycle progression^25^. Overexpression of a dominant negative version of PAKa, PAKa-c, consisting of the CRIB and the kinase domain^26^ had an impact on the adhesive properties of *corB*^−^ and led to reduced adhesion (Table 3). Phagocytosis and development were not affected (Supplementary Table 2).

## Discussion

CRIB domains are found in many effectors of the Rac and Cdc42 GTPases. Upon binding of the activated GTPase the effectors can establish further interactions or deploy catalytic activities. They are present in WASP, PAKs or phospholipase D2 (PLD) the latter carrying less well conserved CRIB domains^27^. The CRIB domains in the coronin proteins also show only a moderate degree of conservation. They only recently received some attention and it was shown for *Dictyostelium* coronin and mammalian Coronin-1C that they preferably bind GDP-loaded Rac proteins. Through this activity they regulate signaling cascades in case of coronin and cell migration in case of Coronin-1C. Coronin regulates the availability of Racs for activation of PAKa which regulates myosin heavy chain kinase, Coronin-1C together with RCC2 sequesters GDP-bound Rac in the cell and prevents its activation^11^,^28^.

Here we studied the CRIB motifs present in the propeller structures of coronin7. The *Dictyostelium* genome encodes several Rac family proteins and they are broadly classified into two groups^15^, a Rac1 family (Rac1a-Rac1c) which shows high similarity to mammalian Racs and a *Dictyostelium* specific sub-family from RacA to RacL. We have previously found that coronin from *Dictyostelium* showed varying affinities for different Rac family members^11^. Similarly, in our pulldown assays CRN7 WT showed a stronger preference for GDP-loaded forms of RacA, RacC, and RacE and bound weakly to Rac1a and Rac1b. By mutational analysis where we exchanged the N-terminally located conserved residues of each CRIB domain we found that both CRIB domains can bind to Rac proteins. In MUT3 in which both CRIBs were mutated the binding was reduced but not completely abrogated. For MUT1 and MUT3 we observed that in several (MUT1) or most cases (MUT3) the preference for the GDP-loaded form was lost and both forms were nearly equally well bound. MUT2 with an intact CRIB1 retained the preference for GDP-Rac for Rac1a and RacE. This indicates that the CRIB motifs of CRN7 (CRIB1 and CRIB2) show a general preference for most Rac family members and also bind preferentially to a specific set of sub-family members. In the CRIB mutants it is the preference for the specific subset of Rac members which is affected retaining the general binding capability. In addition, both CRIB1 and CRIB2 showed variation in the amino acid sequence conservation which might explain the differences in the preference for Rac members.

Coronins are F-actin binding proteins and one possibility is that the Rac protein interaction might interfere with the F-actin interaction. For human Coronin 1B a conserved surface-exposed arginine (R30) in the β-propeller was identified as F-actin binding site^17^. The equivalent residue in Coronin 1A similarly mediated F-actin binding whereas in Coronin 1C it acted in combination with a second F-actin binding site in the unique region located near the C-terminus^29^,^30^. In the *Dictyostelium* coronins the F-actin binding site has not been narrowed down, however, the N-terminal sequences in the β-propeller around the proposed F-actin binding site are well conserved with the arginine changed into a lysine. These residues are however located some distance away from the CRIB domain in the respective β-propellers (Fig. 1ac, enlarged for WD2, double headed arrow). An interference of Rac binding with the F-actin binding therefore does not appear to be very likely. Furthermore, we found that the CRIB mutations did not have an impact on the association of the proteins with the actin cytoskeleton. In Coronin 1C the situation differs since it binds to Rac1 in the proximity of the F-actin binding site^30^.

The potential of the mutant CRN7 proteins to rescue the *corB*^−^ phenotype varied. For cell motility and phagocytosis either one of the CRIB domains had to be intact for re-establishing the wild-type phenotype. Actin dynamics as assessed by actin rod formation after DMSO treatment was rescued to varying degrees by MUT2 and MUT3, whereas rescue of cAMP induced actin polymerization was achieved by wild type and all mutant proteins leaving the CRN7 impact on this process independent of Rac GTPases. Development was reverted to normal by GFP-CRN7 WT and MUT2 indicating a role for CRIB1 (Supplementary Table 1). We tried to link specific Rac proteins to the specific pathways which were affected by CRN7. A review of the available literature showed however, that several Rac proteins function in the same pathways making such an attempt difficult. For instance, Rac1b, RacB and RacC function in cell motility and loss of RacB and RacC led to reduced motility. They also affect F-actin assembly, although CRN7 appears to act independent of Rac GTPases in this process. The Rac1 isoforms, RacB and RacC further play roles in endocytosis and phagocytosis, and the Rac1 isoforms and RacB are important for development^22^,^31^,^32^,^33^,^34^,^35^. In our pull down experiments we found that MUT1 had a high affinity for RacA and RacC and preferred their GDP loaded version. MUT3 showed less efficient binding and in addition had lost the preference for the GDP-loaded proteins. RacE is unique. It regulates cytokinesis and cortical tension^36^,^37^. Although it binds strongly to CRN7 cytokinesis is normal in *corB*^−^.

In the cellular phenotypes studied the downstream effectors of Rac proteins are WASP, SCAR and PAKa. WASP and PAKa harbor CRIB domains that are activated by GTP-bound Rac proteins, SCAR lacks a CRIB domain. Instead, it is a component of a protein complex which is regulated by Rac^38^. We found that ectopic expression of WASP in *corB*^−^ affected phagocytosis restoring wild-type behavior but had no impact on development and cell motility (Supplementary Table 2). Ectopic expression of SCAR resulted in altered development leading to the formation of multiple tips and causing a strong delay. SCAR deficient cells also form multi-tipped structures and show faster development^23^. Additionally, we have found a reduced amount of SCAR protein levels in *corB*^−^ cells suggesting a positive role for CRN7 in SCAR stabilization and activity. The reduced SCAR levels also explain the accelerated development observed in *corB*^−^ cells. Finally, PAKa could be linked to adhesion to a surface as overexpression of a dominant negative version rendered the cells less adhesive than *corB*^−^.

**Figure 6:**
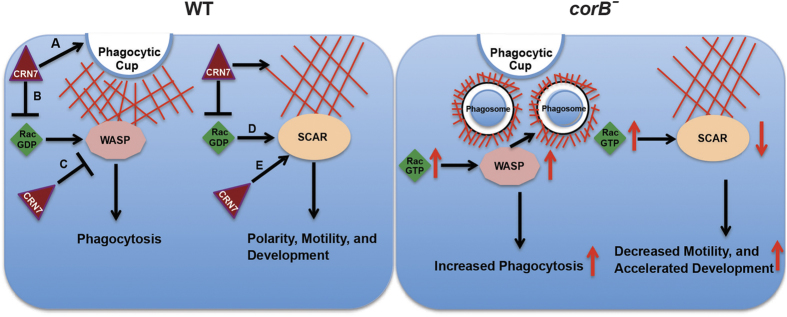
Model for CRN7 mediated regulation of phagocytosis and development through major Rac downstream effectors WASP and SCAR, respectively. In WT cells A) CRN7 regulates actin de-polymerization and prevents phagosome formation (arrow at the tip of the phagocytic cup)^7^,^12^, B) CRN7 prevents ‘local’ activation of Rac GTPases by sequestering Rac-GDP and impacts WASP activation, C) CRN7 interacts with WASP and inhibits its activity (phagocytosis), D) CRN7 mediated inhibition of Rac activity affects SCAR activation, E) CRN7 binds to SCAR and affects its activity thereby regulating motility and development. In CRN7 KO cells (*corB*^−^), absence of CRN7 leads to increased local levels of Rac-GTP. The higher Rac-GTP levels activate WASP resulting in increased phagocytosis. On the other hand loss of CRN7 also results in decreased SCAR activity, which affects motility (decreased) and development (accelerated).

From these data we propose that CRN7 acts upstream of SCAR and WASP. We propose that it keeps Rac GTPases in their GDP-bound form and prevents locally the activation of the downstream targets. Loss of CRN7 leads to “overactivity” of WASP which then activates F-actin assembly leading to increased phagocytosis. CRN7 also stabilizes SCAR and regulates development and cell motility (Fig. 6).

## Methods

### Growth and development of *Dictyostelium* strains

*D. discoideum* strain AX2 was used as wild type strain. *corB*^−^ has been described^12^. Cells were cultured either in petri dishes or in suspension culture (160 rpm) at 22 °C with appropriate antibiotics. All strains were grown and maintained as described, transformation was done by electroporation using plasmids encoding GFP-tagged CRN7^12^, GFP-WASP, GFP-SCAR, GFP-PAKA-c^26^. Selection was with G418 (4 μg/ml). For development logarithmically growing cells were harvested, washed with Soerensen phosphate buffer (17 mM sodium-potassium-phosphate, pH 6.0) and deposited on phosphate agar plates (5 × 10^7^ cells per plate, 10 cm in diameter).

### Generation of GST- and GFP-fusion proteins

Plasmids encoding GFP-tagged CRN7 and GFP- and GST- tagged N (NT)- and C (CT)-terminal WD40 repeat domains of CRN7 have been described previously^12^.

The CRIB domains NT CRIB and CT CRIB in the N-terminal and C-terminal β-propeller, respectively, were targeted for mutation. In GFP-MUT1 the residues LSSA in the NT CRIB were mutated to AASA, in GFP-MUT2 the residues YTTT in the CT CRIB were mutated to AATA by site directed mutagenesis (Stratagene) using sequence specific mutant primers (MUT1 5′GCAATTAGATCAGGTGGCAACATCAGCAGCAAGTGCATCAATTGTTTGTAGTGGACATTCG-3′; MUT2 5′CCGCTAATAAATCAAATAATGCCGCTACAGCCGAGGCAGATTTCGTTGG-3′). GFP-MUT3 carried both mutated CRIB domains (MUT1+MUT2). GFP-CRN7 WT and CRIB mutants (GFP-MUT1, GFP-MUT2, and GFP-MUT3) were then transformed into *corB*^−^ cells using electroporation and mutant colonies expressing GFP-fusion proteins were selected with G418 (4 μg/ml). DdWASP and Scar cDNA sequences were cloned into a pDEX-GFP plasmid or pDGFP-MCS Neo. GFP is fused to the N-terminus of the proteins. Expression was under the control of the constitutively active actin15 promoter. Selection of *D. discoideum* transformants was with G418 (4 μg/ml).

To generate RacC mutations the following primers were used: RacC Mut1 For GCAAACAATCGTTTCCCAAAAAAATATATTCCAACTGTATTCG, RacC Mut1 Rev CGAATACAGTTGGAATATATTTTTTTGGGAAACGATTGTTTGC; RacC Mut2 For GATTATATTCCAACTGTATTCGCTAATTATGTTGTAAATCTTACAGC, RacC Mut2 Rev GCTGTAAGATTTACAACATAATTAGCGAATACAGTTGGAATATAATC; RacC Mut3 For CCAACTGTATTCGATAATAAAGTTGTAAATCTTACAGC, RacC Mut3 Rev GCTGTAAGATTTACAACTTTATTATCGAATACAGTTGG; RacC Mut4 For GGGATACTGCAGGTCAAAAAAAGTACGATAAATTAAGACC, RacC Mut4 Rev GGTCTTAATTTATCGTACTTTTTTTGACCTGCAGTATCCC; RacC Mut5 For GGTACTAAATTAGATACACGTCAACAAAGAGGTGTTTTAGATAAACTTC, RacC Mut5 Rev GAAGTTTATCTAAAACACCTCTTTGTTGACGTGTATCTAATTTAGTACC.

### CRN7-Rac interaction assays, pull down experiments and immunoprecipitations

GST, GST-RacA, C, E, 1a and 1b fusion proteins were expressed in *E. coli* BL21 and purified from the soluble fraction using Glutathione Sepharose affinity columns (GE Healthcare). The purified proteins were loaded with GDP or GTPγS (2 mM each) in nucleotide exchange buffer (25 mM Hepes, pH 7.4, 100 mM NaCl, 10 mM EDTA, and 1 mM DTT) for 1 h at 4 °C. For analysis of the interaction of CRN7 proteins with Rac GTPases, *corB*^−^ cells (5 × 10^7^ cells) expressing GFP-CRN7, GFP-CRN7 MUT1 (MUT1), GFP-CRN7 MUT2 (MUT2), and GFP-CRN7 MUT3 (MUT3) were lysed by sonication in lysis buffer (25 mM Tris-HCl, pH 7.5, 100 mM NaCl, 5 mM MgCl 2, 2 mM EGTA, 2 mM DTT, and 1% Nonidet P-40 with EDTA-free protease inhibitor mixture (Roche)), and equivalent amounts of cell lysates were added to Glutathione Sepharose 4B beads (GE Healthcare) carrying Glutathione S-Transferase (GST) and GST tagged fusion proteins preloaded with either GDP or GTPγS.

For immunoprecipitation cells (5 × 10^7^) expressing the appropriate proteins were lysed by pipetting several times in 500 μl lysis buffer (10 mM Tris-HCl, pH 7.5, 100 mM NaCl, 1 mM EDTA, and 0.5% NP40 with protease inhibitor cocktail) and incubated on ice for 20 minutes. 500 μl of dilution buffer (10 mM Tris-HCl, pH 7.5, 100 mM NaCl, and 1 mM EDTA) was then added to the clarified lysate and incubated with 20 μl of GFP-TRAP beads (ChromoTek, Martinsried, Germany) for 2 hours at 4 °C. The beads were washed and the precipitates analysed by western blotting with anti-GFP mAb K3-184-2^39^. Alternatively cells were lysed in lysis buffer containing 20 mM Tris-HCl, pH 7.5, 150 mM NaCl, 2% glycerol, 2 mM EDTA, 0.5% NP40, protease inhibitor cocktail (Sigma), 1 mM DTT). Pull downs were performed with Glutathione Sepharose 4B beads (GE Healthcare) carrying GST tagged fusion proteins, immunoprecipitations were done with Protein A-Sepharose 4B beads (Sigma) carrying appropriate antibodies. After incubation at 4 °C for 2 hours, beads were gently pelleted and washed with lysis buffer containing 0.25% NP40. Samples were boiled and the proteins analyzed by western blotting with appropriate antibodies. For control, GST and beads were used. In general, the experiments were carried out three times.

In order to determine the levels of activated Rac in *D. discoideum* cells, a pull down with the GTPase-binding domain (GBD) from rat PAK1 kinase (GST-PAK-GBD) which specifically interacts with the GTP-bound form of Rac1 was carried out followed by western blot analysis with polyclonal antibodies against Rac as described^11^,^40^. The blots were analysed and the amount of activated Rac from AX2 and *corB*^−^ cells calculated by image J.

### Antibodies used in western blots and immunofluorescence

mAb K67-31-5 is directed against the PST domain of *Dictyostelium* CRN7^12^, mAb 176-3-6 detects coronin^16^, mAb 135–409 recognizing cap34^41^ was used for normalization, sheep anti SCAR was obtained from Dr. C. Saxe^42^, mAb 56-396-5 recognizes myosin heavy chain^43^, mAb act1 is specific for actin^44^, mAb K3-184-2 is specific for GFP^39^. Polyclonal GST-specific antibodies detected GST and GST fusion proteins.

### Mutant analysis

Immunofluorescence was performed as described^45^. Cells were fixed by ice cold methanol (5 min, –20 °C) and stained with the appropriate antibodies. Fixed cells and living cells were imaged using confocal laser scanning microscopy (TCS SP5 Leica). DMSO treatment, phagocytosis and cell motility assays were performed as described^7^. Specifically, for analysis of actin rod formation after DMSO treatment cells were incubated in growth medium in the presence of 5% DMSO for 10 min at room temperature, fixed with ethanol and immunolabeled for actin with mAb act-1. Nuclei were stained with DAPI. Analysis was carried out by confocal microscopy. Between one and two hundred cells were analysed. For analysis of cell adhesion 2 × 10^6^ cells were incubated in a six-well plate at 22 °C for 4 hours, then the plates were shaken on a gyratory shaker at 100 rpm for one hour. The detached cells were counted in a hemocytometer. The total number of cells was determined after resuspension of the cells and the percentage of detached cells calculated. The results were calculated from more than ten experiments. For cell size determination cells were transferred to Soerensen phosphate buffer which contained 20 mM EDTA. Development was analyzed by plating 5 × 10^7^ cells on a phosphate agar plate (10 cm in diameter). Pictures were taken at the indicated time points. Triton-insoluble cytoskeletons were prepared as described^12^, for F-actin accumulation after a cAMP stimulus (10^−7^ M) cells were kept in Soerensen phosphate buffer in shaken suspension at a density of 2 × 10^7^ cells/ml for 6 hours^12^.

Transcript levels were was quantified by qRT-PCR in RNA samples from growth phase cells using coronin and CRN7 specific primers (coronin 5′ TTGCTTTCCACCCATTCAATGAAAACC; 3′ CGGCAACATTATCAGCAACTGGATTGA; CRN7 5′ CGCTCGTTTCTATCGTTCAACCATTC; 3′ CTTCTTTCTTTGGTGGTGGTGGAGCAT.

For statistical evaluation the Student’s t test was used.

## Additional Information

**How to cite this article**: Swaminathan, K. *et al.* Coronin7 regulates WASP and SCAR through CRIB mediated interaction with Rac proteins. *Sci. Rep.* **5**, 14437; doi: 10.1038/srep14437 (2015).

## Supplementary Material

Supplementary Information

## Figures and Tables

**Table 1:**
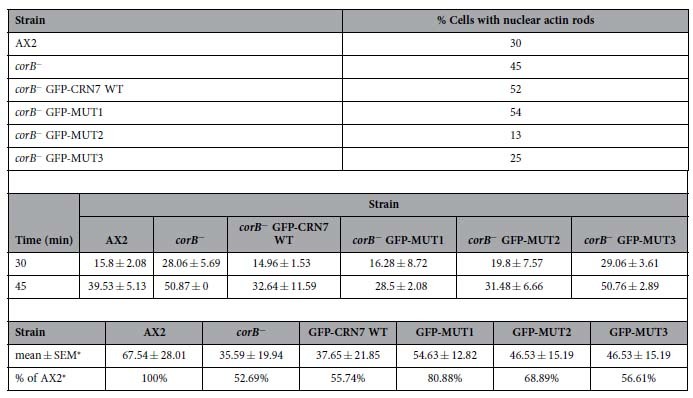
Rescue activities of CRN7 and mutant proteins in *corB* ^−^ on actin rod formation, phagocytosis and adhesion to a plastic surface.

*Actin rod formation upon DMSO treatment.* Cells were treated with DMSO (5%) and fixed with methanol after 10 minutes of incubation. Staining was with mAb act1 for actin and DAPI for nuclei. Between 100 and 200 cells were analyzed per experiment. The differences were statistically significant (P < 0.05).

*Phagocytosis of *corB*^−^ and *corB*^−^ cells expressing the indicated CRN7 proteins*. Cells were incubated with yeast cells and fixed at the indicated time points. Staining was with mAb act1 to visualize the cells. The data represent the mean from three indivdual experiments with 180, 180 and 400 cells evaluated each. Mean and SD are shown. The difference between AX2 and *corB*^−^ and *corB*^−^ GFP-MUT3 was statistically significant (P < 0.05). Care was taken to analyze cells expressing comparable levels of the GFP-fusion proteins.

*Adhesion of CRN7 mutants to a plastic surface*. The number of detached cells was determined after one hour of shaking at 100 rpm and set into relation to the total number of cells. The data are from 3 independent experiments in which each assay was performed in triplicate.

^*^Detached cells.

**Table 2:** Analysis of random motility.

	AX2	*corB*^−^	*corB*^−^ GFP-CRN7WT	*corB*^−^ MUT1
Speed (μm/min)	4.46 ± 2.52	2.87 ± 1.28	5.41 ± 1.24	4.91 ± 1.06
Direction change (deg)	67 ± 13.45	67.81 ± 10.73	43.39 ± 13.36	41.01 ± 19.76
Persistence (μm/min-deg)	0.73 ± 0.46	0.56 ± 0.31	1.3 ± 0.49	1.39 ± 0.78

	***corB***^**−**^ **MUT2**	***corB***^**−**^ **MUT3**	**AX2 WASP**	***corB***^**−**^ **WASP**
Speed (μm/min)	5.49 ± 2.67	3.51 ± 0.76	3.03 ± 1.26	3.22 ± 1.0
Direction change (deg)	55.45 ± 10.08	59.11 ± 7.15	69.67 ± 14.97	58.93 ± 13.25
Persistence (μm/min-deg)	1.21 ± 0.88	0.68 ± 0.2	0.55 ± 0.34	0.74 ± 0.33

Images were taken at 10 × magnification every 30 s. In all cases cells were recorded over a period of 30 min. Cells were recorded and after tracing of the cells the centroid of the cells was determined by computer-assisted analysis (DIAS). This allows calculations of speed, direction change and persistence. Direction change represents the average change of direction (in degrees) between two subsequent images. Persistence is an estimation of movement in the direction of the path. Values are mean ± standard deviation.

**Table 3:**
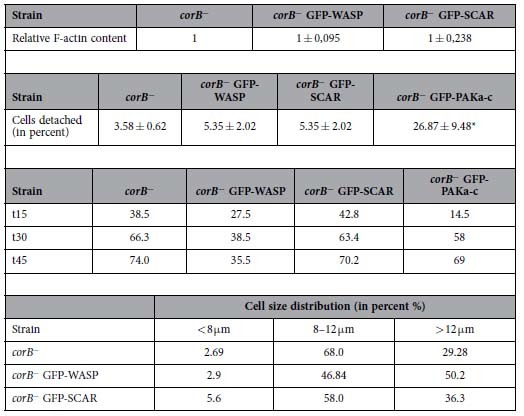
Effect of WASP, SCAR and PAKA-c expression in *corB*^**−**^ on F-actin content, cell substrate adhesion, phagocytosis and cell size.

*F-actin content*. Growth phase cells were harvested and Triton-insoluble pellets prepared. The F-actin amount of *corB*^−^ was set to 1. The results are from three independent experiments.

*Effect on adhesion to a plastic surface*. The results are from five different experiments. Each experiment was carried out in triplicate. ±SD; *statistically significant, P < 0.001.

*Effect on phagocytosis*. The total number of cells that have taken up yeast particles at the indicated time points (min) are given in percent. The data are from three independent experiments with at least 200 cells analyzed.

*Effect on cell size*. Cells were kept in Soerensen phosphate buffer in the presence of 20 mM EDTA. Between 250 and 1000 cells were analyzed.
