# Supplementary material for: Coronin7 regulates WASP and SCAR through CRIB mediated interaction with Rac proteins
Source: Sci Rep. 2015 Sep 28;5:14437. doi: 10.1038/srep14437 (PMC4585930; doi:10.1038/srep14437)
Supplement: Supplementary Information [file srep14437-s1.pdf]

Supplementary Information

Coronin7 regulates WASP and SCAR through CRIB mediated interaction with Rac proteins

Karthic Swaminathan, Maria Stumpf, Rolf Müller, Anna-Carolin Horn, Julia Schmidbauer, Ludwig Eichinger, Annette Müller-Taubenberger, Jan Faix, Angelika A. Noegel

Supplementary Tables

Supplementary Table 1. Rescue potential of Mutant CRN7 proteins.

Phenotype	AX2	*corB*$^{-}$	CRN7 WT	MUT1	MUT2	MUT3
Motility	WT	reduced	WT	WT	WT	like *corB*$^{-}$
cAMP induced F-actin assembly	WT	enhanced first peak	WT	WT	WT	WT
Actin rod formation 10 min (%)	WT 30%	enhanced 45%	enhanced 52%	enhanced 54%	lower than WT 12.8%	WT 25.4%
Phagocytosis	WT	enhanced	WT	WT	WT	like *corB*$^{-}$
Adhesion	WT	enhanced as compared to AX2	like *corB*$^{-}$	closer to WT	closer to WT	like *corB*$^{-}$
Development	WT	enhanced	like AX2 (WT)	like *corB*$^{-}$	slower, closer to WT	like *corB*$^{-}$

Supplementary Table 2. Rescue activities of WASP, SCAR, PakA-c in *corB*$^{-}$.

Phenotype	*corB*$^{-}$	*corB*$^{-}$ GFP-WASP	*corB*$^{-}$ GFP-SCAR	*corB*$^{-}$ PAKa-C
Cell motility	reduced	reduced, not different from *corB*$^{-}$	not tested	not tested
Adhesion in percent	enhanced as compared to AX2	like *corB*-	like *corB*$^{-}$	less adhesive than *corB*$^{-}$
Yeast Phagocytosis	enhanced as compared to AX2	like AX2	like *corB*$^{-}$	not tested
Development	faster as compared to AX2	like *corB*$^{-}$	delayed; multiple tips $^{-}$	like *corB*$^{-}$
Total F-actin content	1	1.03±0.1, like *corB*$^{-}$	0.99±0.24 , like *corB*$^{-}$	not tested
Cell size	similar to AX2	increased as compared to *corB*$^{-}$	comparable to *corB*$^{-}$	not tested

Legends to Supplementary Figures

Supplementary Figure S1. Comparison of CRN7 and coronin in Rac binding assays. GST tagged RacC and RacE were loaded with GDP or GTPγS and pulldown assays performed with lysate from *corB*⁻ cells expressing GFP-CRN7 WT. The resulting blot was probed for the presence of GFP-CRN7 WT at 130 kDa with mAb K3-184-2 and coronin at 55 kDa with mAb 176-3-6. I, input lysate.

Supplementary Figure S2. Binding of CRN7 and CRIB mutants to various Rac proteins. (a) Binding of MUT1 to various Rac proteins. GST tagged Rac proteins were loaded with GDP or GTPγS and used to precipitate GFP-MUT1. Absolute intensities are shown in order to demonstrate the different interaction strengths. (b) Binding of CRN7 and CRIB mutants to Rac proteins as indicated. The data are mean values obtained from several experiments. Relative intensities (in percent) are shown.

Supplementary Figure S3. Association of CRN7 CRIB mutant proteins with the actin cytoskeleton. Triton X-100-insoluble cytoskeletons were prepared from cells expressing the various GFP-tagged proteins and supernatant and pellet fractions analyzed by western blotting using mAb K3-184-2. Equivalent amounts were loaded. In case of MUT1 five times higher amounts of the supernatant fraction was loaded.

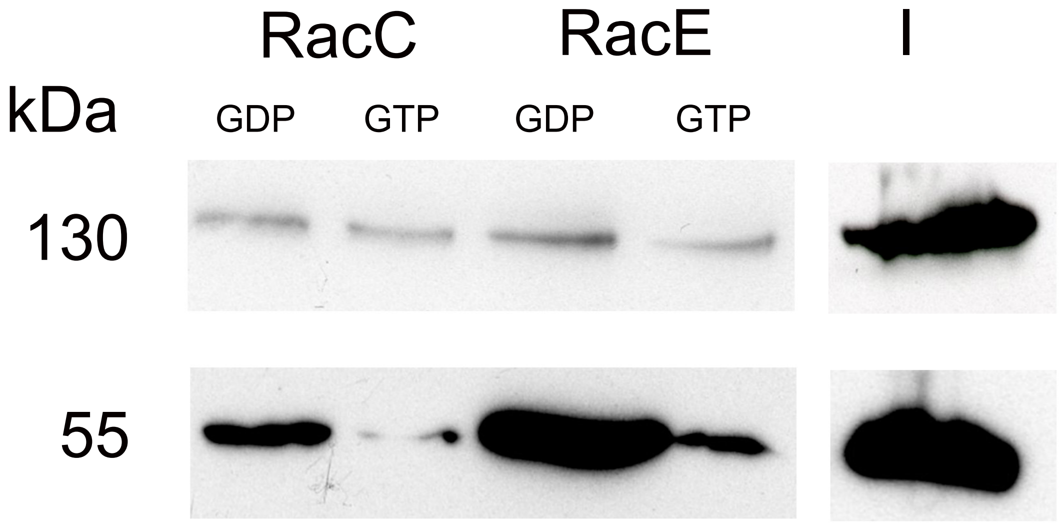

Figure S1

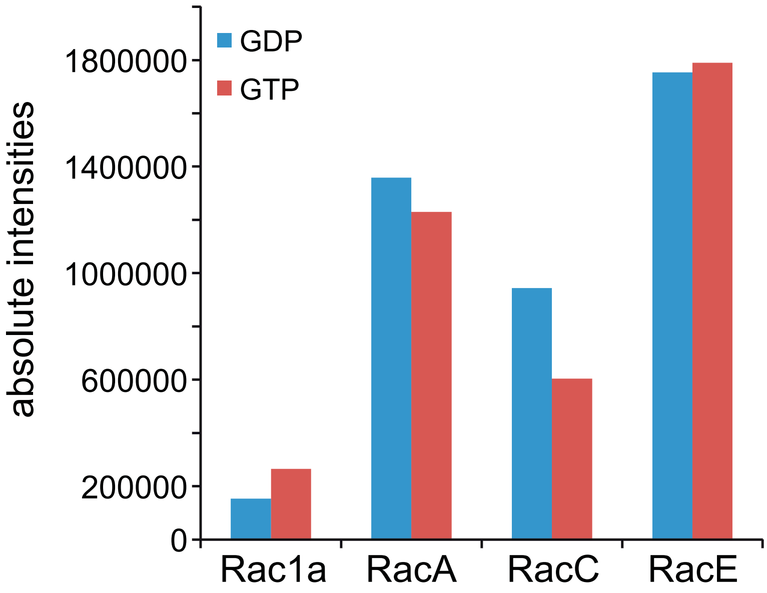
GDP
GTP
absolute intensities
1800000
1400000
1000000
600000
200000
0
Rac1a
RacA
RacC
RacE

Figure S2a

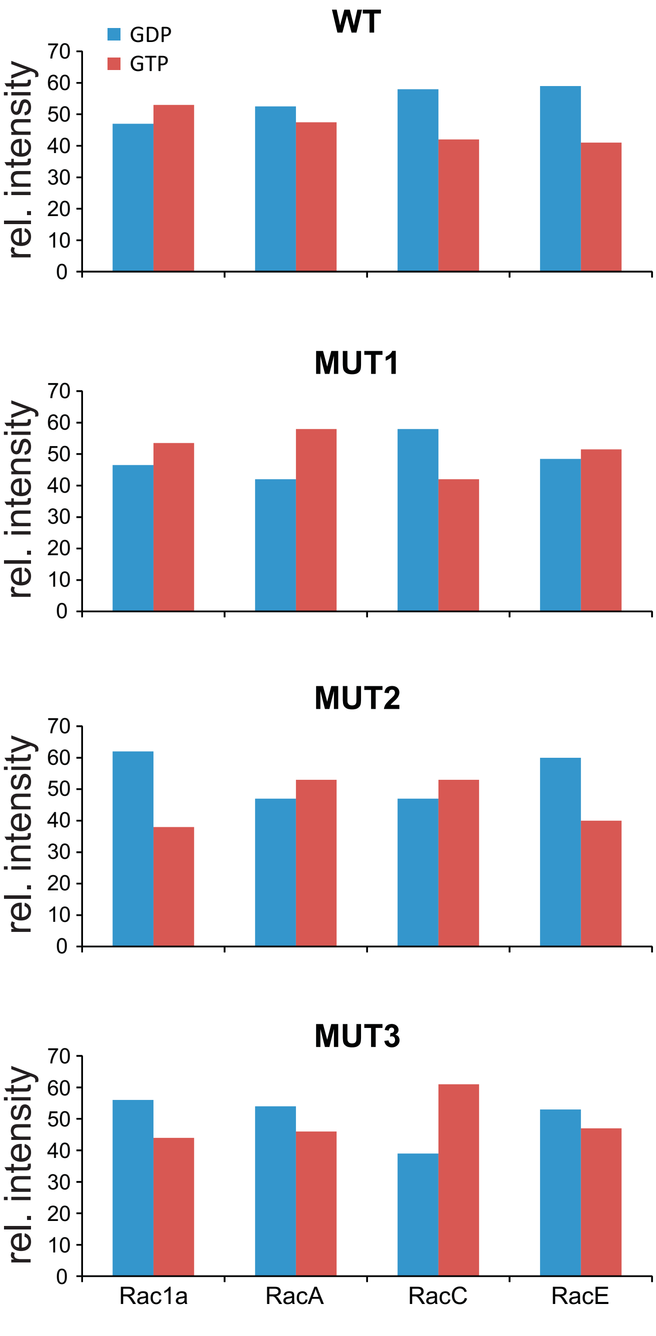
WT
GDP
GTP
rel. intensity
0
10
20
30
40
50
60
70
MUT1
MUT2
MUT3
Rac1a
RacA
RacC
RacE

Figure S2b

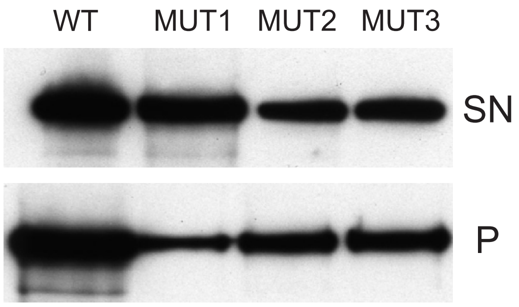

Figure S3
